# Elastoplastic Mechanical Properties and Kinematic Hardening Model of 35CrNi3MoVR

**DOI:** 10.3390/ma17133223

**Published:** 2024-07-01

**Authors:** Zhao Zhang, Xuesheng Wang, Qinzhu Chen

**Affiliations:** Key Laboratory of Safety Science of Pressurized System, Ministry of Education, East China University of Science and Technology, 130 Meilong Road, Shanghai 200237, China; zzhang1992@126.com (Z.Z.);

**Keywords:** elastoplasticity, constitutive model, Bauschinger effect, autofrettage, residual stress, kinematic hardening model, ultra-high pressure

## Abstract

The existing tensile–compression elastoplastic models are not suitable for varies of materials. An accurate constitutive model of the elastoplastic mechanical properties more suitable for 35CrNi3MoVR was produced by optimizing the existing fitting equations based on uniaxial tensile–compression tests, which are able to describe the elastoplastic stress–strain relation and Bauschinger effect varying with the maximum tensile plastic strain. A UMAT subroutine of the constitutive model in ABAQUS was proposed and conducted for FEM calculation. Hydraulic autofrettage tests were carried out under different pressures on thick-walled 35CrNi3MoVR tubes, and the results were compared with those of FEM calculations to further validate the accuracy of the fitting model. The results show that the constructed power function kinematic hardening model can effectively describe the elastoplastic mechanical properties of 35CrNi3MoVR and can be applied to the autofrettage calculation of this material. The comparison among the calculation results of different models proved that the model proposed in this research has better performance compared to other existing models. Taking the Mises stress at the inner surface of the thick-walled tubes as the evaluation criterion, the error of the power function kinematic hardening model reaches less than 3%, decreasing the error by at least 50%.

## 1. Introduction

The autofrettage of ultra-high pressure thick-walled pressure vessels is widely applied in various fields, such as aerospace, the petrochemical industry, and nuclear energy. The implementation of self-strengthening technology can effectively solve the issue of uneven stress distribution caused by excessive working pressure; additionally, it avoids material consumption and processing difficulties due to excessively thick walls, and it improves the corrosion resistance of the structure. The autofrettage process involves applying internal pressure or another form of deformation load on the inner surface of the thick-walled structure, causing the material near the inner wall to yield. Upon the removal of the load, the elastic zone attempts to revert to its original position, while the plastic zone tends to maintain a permanent deformation state. The elastic contraction of the outer material results in the compression of plastically deformed material in the inner layer. When the container is subjected to a working load, the stress distribution along the wall thickness tends to be uniform [1,2,3,4]. A variety of processes can be employed to achieve autofrettage, which can be classified as hydraulic autofrettage, molded autofrettage, explosive autofrettage, thermal autofrettage, or rotational autofrettage, depending on the type of forming load [5,6,7]. Hydraulic autofrettage is one of the most traditional and widely employed autofrettage methods [8,9]. A substantial corpus of theoretical, finite element calculations and experimental research has been conducted to explicate this method. Sergei Alexandrov [10] proposed a semi-analytic solution considering the impact of the Bauschinger effect using a material model that accounts for the response of typical high-strength steel, and the results were consistent with numerical solutions. Shim W. S. [11] accurately predicted the residual stress of SNCM 8 high-strength steel using the Kendall model, where a constant Bauschinger effect factor was used, and the results were compared with those of analytical and finite element analysis. Hakan Çandar [12] measured radial and hoop residual stresses induced by autofrettage processes using X-ray diffraction methods, along with FEM calculations of 2D axisymmetric analysis. Yanling Ma [13] quantitatively revealed the residual strain distributions, positions of elastic–plastic juncture and first yielding, etc., of autofrettaged thick-walled pressure vessels using the finite element method, analytical calculations, and neutron diffraction. M. Maleki [14] evaluated residual stress distributions in autofrettaged homogeneous spherical pressure vessels subjected to different autofrettage pressures.

The Bauschinger effect refers to the phenomenon whereby the yield strength of a metallic material decreases when loaded in the opposite direction, which significantly impacts the effectiveness of the autofrettage process. Due to strain hardening and the Bauschinger effect, most materials do not show ideal elastoplastic characteristics, so the description of the elastoplastic properties of the actual material requires the application of a simplified model, the widely used forms of which are the linear strain hardening model, the power strain hardening model, or a combination of the two. The Bauschinger effect of materials will influence the residual stress on the inner surface after autofrettage, and the residual stress at this position substantially impacts the assessment of autofrettage effects [15,16]. Milligan R. V. [17] quantitatively evaluated the Bauschinger coefficient at different tensile overstrain degrees of modified 4330 steel under different heat treatment states, discovering that the Bauschinger effect decreased with the increase in tensile plasticity, but it remained approximately unchanged after the tensile plasticity reached ~2%. Megahed M. M. [18] employed a uniaxial tensile–compression test to develop an elastoplastic constitutive model that incorporates the Bauschinger effect of the material. This effect entails an increase in the compressive yield stress in relation to the maximum tensile strain. Additionally, the elastoplastic relationship of the tensile section is simplified to a bilinear model, the compressive plastic modulus is considered to be equivalent to the tensile modulus of elasticity in the model, and the stress–strain relationship of the compressive plastic section at different degrees of maximum tensile strain is also represented by a settled nonlinear relationship. Troiano and Parker A.P. [19] conducted uniaxial tensile–compressive tests on A723, HY80, and PH 13-8mo, and the corresponding tensile–compression data were provided. Huang X. P. [20] proposed a generalized autofrettage model that incorporates the strain hardening relationship of the material and the Bauschinger effect based on the actual tensile–strain relationship of 30CrNiMo8. However, the variation in the Bauschinger effect with the change in maximum tensile strain was not taken into account in the calculations, as the tensile–compression relationship of the 30CrNiMo8 material at the approximate maximum tensile strain was adopted. Rolf R. de Swardt [21] employed a bilinear model to calculate the autofrettage process numerically, in which the variations in the Bauschinger effect and the compressive plastic slope caused by the variation in the maximum tensile strain were considered. However, there was a certain deviation from the actual material compression curve, due to the bilinear simplification. Parker A.P. [22,23,24,25] proposed a revised kinematic hardening model considering the variation in the Bauschinger effect. The fitting equation form given was suitable for the strain hardening materials tested and was able to accurately describe the elastoplastic mechanical relationships during the loading and unloading processes under different maximum tensile strains. The prediction accuracy of this method was much higher than that of the ideal elastoplastic model. In fact, this kinematic hardening model is a kind of variable material properties method [26]. E. Troiano [27] studied the mechanisms of HB7 steels and fitted elastoplastic characteristics similar to those of Parker A.P.’s method. G. H. Farrahi et al. [28] concluded that an accurate material behavior model plays an important role in estimating the residual stresses of the autofrettage tubes, and a modification of the Chaboche hardening evolution equation was employed to describe the nonlinear characteristics of the material. Troiano E. et al. [29], Gibson M. C. [30], and Hu Z. [31] conducted finite element simulations with various autofrettage modes based on the constitutive elastoplastic material model established by Parker A.P., and the results further validated the accuracy of the methodology. Zhong Hu [32], based on Parker A.P.’s A723 data and using a data processing method similar to Huang. X. P.’s, conducted numerical simulation research on the extrusion autofrettage process of thick-walled straight lines. Loffredo M. [33] conducted a set of tensile–compressive tests on AISI 4140 steel, derived a multiaxial elastoplastic model from the combination of nonlinear kinematic hardening and nonlinear isotropic softening, and proposed a fitting method for the model parameters, which was finally implemented in ANSYS software. M. Molaie et al. [34] employed a more simplified mathematical expression to represent the nonlinearity observed in tensile–compression testing, which is more effective in capturing the elastoplastic curve of materials exhibiting a plastic plateau phenomenon. However, this model is limited in its ability to describe the stress–strain curve of the material under a specific degree of maximum tensile plastic strain. Yan Li [35] used the ideal elastoplastic model to numerically calculate the autofrettage of high-pressure seamless steel cylinders for hydrogen, the results were used for fatigue life analysis. Shufen [36] numerically investigated the process of thermally assisted rotational autofrettage of long cylinders with free ends, with the characteristics of the material varying with temperature, where the simplest ideal elastoplastic model was adopted. Zhong Hu [37] numerically investigated the autofrettage and re-autofrettage of fluid end blocks using a true material model, where more accurate results were obtained compared with the bilinear kinematic hardening material model’s simulation results.

The preceding literature indicates that Parker A.P.’s fitting method, for instance, appears to be the most prevalent and has seen widespread application; however, it is necessary to acknowledge that it still possesses certain limitations. It is challenging to develop a universal equation that can accurately describe the intrinsic tensile–compressive elastoplastic models of different materials, even for materials with similar properties. Consequently, in order to provide a more accurate description of the true stress–strain properties of a specific material, it is necessary to conduct experimental and numerical studies on their elastoplastic constitutive relationships. This study aimed to develop a revised kinematic hardening model composed of a group of equations that can be used to accurately describe the elastoplastic mechanical properties of 35CrNi3MoVR, which is a kind of steel used for ultra-high pressure vessels. A group of uniaxial mechanical property tests were carried out to determine the stress–strain relationship of the material during the process of loading and unloading. After that, a group of equations were used to fit the elastoplastic testing data. The logic used for the finite element method calculation describing the process from loading to unloading is provided here, and the FEM method is used first for checking the fitting functions and then for simulating the autofrettage process. Autofrettage experiments were performed, and the measured stresses were compared to the FEM results calculated using a series of models, in order to further identify the accuracy of the fitting functions.

## 2. Materials and Methods

### 2.1. Introduction of the Experimental Material

The GB/T 34019-2017 Ultra-high Pressure Vessel is China’s ultra-high pressure vessel standard, involving two different steel grades, of which 35CrNi3MoVR is more commonly used [38]. Currently, there is no research on the Bauschinger characteristics of 35CrNi3MoVR, and the elastoplastic characteristics of the material are still deficient. It is obvious that the use of accurate material models leads to accurate stress calculation results, and the leakage of the elastoplastic characteristics of the material leads to inaccurate stress calculation results for the autofrettage process. This makes it important to research the Bauschinger and elastoplastic characteristics of 35CrNi3MoVR steel.

The raw material for the samples was produced by Inner Mongolia Northern Heavy Industries Group Co., Ltd. in Baotou, China; it is a kind of calm steel manufactured using an electric furnace or converter smelting and processed through off-furnace refining (including vacuum treatment) and electroslag remelting. The forging performance heat treatment consists of normalizing, quenching, and tempering, where the tempering temperature is no less than 540 °C. The performance and heat treatment status of the materials used in this study meet the aforementioned requirements.

The composition and fundamental mechanical properties of 35CrNi3MoVR are presented in Table 1 and Table 2, respectively. Some material suppliers assert that this material is consistent with A723 GR.2, yet, in fact, there are certain differences between the two materials in terms of composition and mechanical properties.

**Figure 1:**
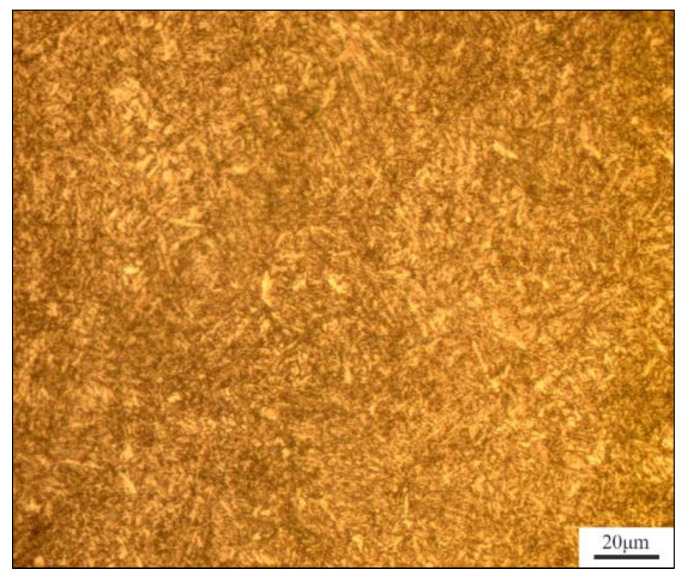
Metallographic microscope image of 35CrNi3MoVR (1000×).

Figure 1 depicts the metallographic diagram of 35CrNi3MoVR; its metallographic organization is characterized by a fine and neat structure, with the majority of the material comprising tempered Sohnite. This indicates that the heat treatment state of the material is favorable.

### 2.2. Explanations of the Uniaxial Mechanical Property Tests

Uniaxial mechanical property testing is a conventional method for studying the mechanical properties of materials, with advantages such as intuitiveness and efficiency, as well as the simplicity of the equipment and operations. Therefore, researchers frequently utilize uniaxial mechanical property testing to investigate the elastoplastic mechanical properties of materials.

The elastoplastic mechanical properties of the material measured differed slightly with the sequence of tension and compression in the uniaxial mechanical properties test. When this material is employed in an autofrettage process, the stress state of the plastic region experiences a transition from tensile elasticity to tensile plasticity, then subsequently to compressive elasticity, and finally to compressive plasticity. During the autofrettage process, the load form of the plastic region is tensile stress, and it becomes compressive stress after autofrettage. This transition of the stress state of the plastic zone during and after autofrettage is consistent with that in uniaxial tension–compression testing. Therefore, uniaxial tensile–compression tests can be used to study the elastoplastic mechanical properties of materials.

In order to conduct uniaxial tensile and compressive mechanical tests of 35CrNi3MoVR steel, it is first necessary to determine the tensile and compressive strains to be achieved by the material under examination. According to the stress conditions of the actual ultra-high pressure autofrettage tubes, it is almost impossible for the total tensile strain to exceed 4.0%, so 4.0% was taken as the maximum total tensile strain in this study. Although the stress condition of the plastic zone is compressive stress, it will not reach a large extent, and it is unlikely to reach a significant degree of negative strain, so the maximum compressive strain can be set at a value slightly lower than 0. According to the mechanical properties of similar materials, when the tensile plastic strain is low, the Bauschinger effect of the material will change significantly. Therefore, the maximum tensile strain value chosen in this test in the lower-strain region was more intensive, while in the higher-strain region it was sparser.

**Figure 2:**
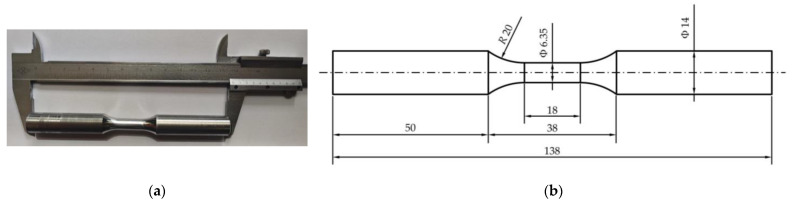
Uniaxial tensile–compression test specimen (**a**) and its parameters (**b**).

The specimen structure and dimensions are depicted in Figure 2; the unit of the parameters in Figure 2b is millimeters.

The testing machine utilized in the uniaxial tensile and compressive mechanical tests was the Zwick/Roell Z330 RED universal testing machine. The stress–strain data of the specimens during the experiment were collected automatically using a Vishay electronic extensometer. The testing details were carried out based on ISO 6892-1:2019. To ensure the accuracy of the test results, three sets of tests were conducted for each test point, and the stress–strain results were averaged.

Tensile–compression tests were conducted in accordance with the total tensile strain degrees specified in Table 3.

## 3. Construction of the Constitutive Modeling

### 3.1. Processing of Experimental Data

The test data were automatically recorded using an electronic extensometer, which provided the engineering stress–strain data of the material. These first needed to be converted to true stress–strain data. For tensile or compression curves of real materials, it is challenging to identify the actual yield point. The accuracy of the constitutive model is highly dependent on the method employed to determine the yield point. In this study, the tensile yield point and compressive yield point were selected as follows:

In the case of the tensile yield point, the existence of a clear elastoplastic cutoff point allows for the direct use of the elastoplastic nonlinear turning point in the actual stress–strain relationship, rather than the use of a simplified method of description, thereby improving the accuracy.

It is challenging to distinguish accurately between the elastic rebound and compression plastic intervals in the unloading process. Furthermore, the experimental results demonstrate that no compressive true stress–true strain curve exhibits a strictly elastic interval, even when the elastic rebound section is considered. The objective of fitting the experimental data in this study was to obtain an ontological relationship as close as possible to the actual material tensile–compression curve, so the plastic yield point methods commonly used in engineering, such as *R*_p0.2_ or *R*_t0.5_, are too large in error and, therefore, unsuitable for use as a way of selecting the compression yield point. For the aforementioned reasons, a straight line was imagined between the maximum stretching point and the point where the stress is zero, which was taken as the strictly elastic interval in the unloading process, and then *R*_p0.05_ was taken for defining the unloading yield point. The slope of the connecting line of the maximum stretching point and the unloading yield point was taken as the unloading elasticity modulus.

**Figure 3:**
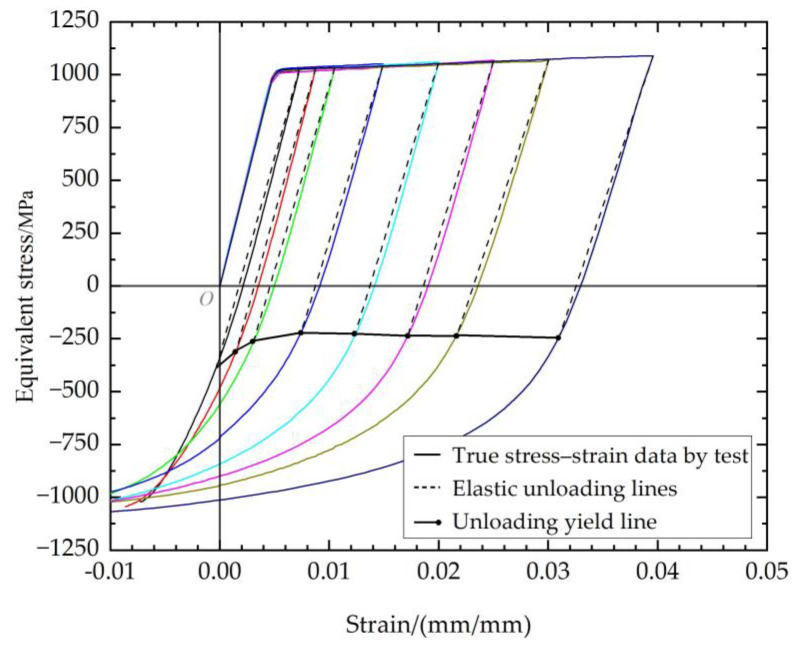
True stress−strain curve and unloading plasticity point.

According to the above method, the stress and strain of the unloading yield point were obtained and displayed in the true stress–strain curve graph at different tensile strains. The unloading yield points are connected by lines with a dot at their ending, which represent the identification of the unloading yield line of the material, as illustrated in Figure 3.

In order to simplify the modeling and calculation process, the expression of the stress–strain data corresponding to different total tensile strains can be formulated based on the corresponding plastic strains [39]. Therefore, it is necessary to transform the material stress–strain data for plastic zones into stress–plastic strain data.

### 3.2. Fitting of the Equations

**Figure 4:**
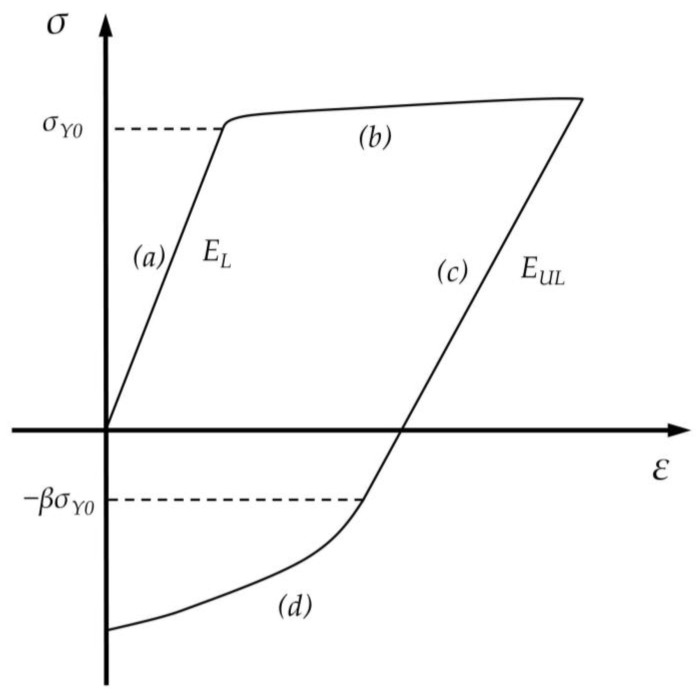
Division of tensile−compression curves and schematic diagrams of the Bauschinger coefficient *β*, loading modulus of elasticity *E_L_*, and unloading modulus of elasticity *E_UL_*.

The tensile–compression curves were divided into different regions, as follows: (a) loading elastic region, (b) loading plastic region, (c) unloading elastic region, and (d) unloading plastic region. The division is shown in Figure 4. The loading plastic region (b) is a curve that begins at the tensile yield point, regardless of the maximum tensile plastic strain. In contrast, the stress–strain relationship for the compressive regions (c) and (d) varies with the maximum tensile plastic strain. Therefore, a functional approach is required to express this relationship, with the maximum tensile plastic strain as the independent variable. 

Various methods currently exist to describe the Bauschinger effect. For simplicity in modeling in this paper, the Bauschinger coefficient *β* was defined as the ratio of compressive yield stress $\sigma_{Y}^{p}$ to tensile yield stress $\sigma_{Y0}$. That is,

(1) $$\beta =\frac{\sigma_{Y}^{p}}{\sigma_{Y0}}$$

In this paper, based on the revised kinematic hardening model proposed by Parker A.P. [25], a more suitable kinematic hardening model is proposed for the mechanical properties of this material, as follows:

For the (a) loading elastic region and (c) unloading elastic region, the stress–strain relationships are linear. Consequently, it is only necessary to express the tensile and compressive elastic moduli in terms of *E_L_* and *E_UL_*, respectively, and *E_UL_* is expressed as a function of the tensile plastic strain in order to express their stress–strain relationships effectively.

For the loading plastic region (b), due to its rapid nonlinear increase in stress in the lower range of plastic strain and its near-linear increase in stress in the higher range of plastic strain, the function proposed by Parker A.P., which was obtained by superimposing and combining the nonlinear relationship of a linear function and the *tanh*( ) function, can effectively describe the relationship in all strain ranges. This equation form has great advantages in fitting accuracy for the loading elastic region, so it was used here. Of course, because of the simplicity of the equation form, power functions are often used to describe the tensile plasticity region; therefore, the power function fitting method is also used here for comparison with that of Parker A.P.

For the unloading plastic region (d), since there is a clear nonlinear relationship in the stress–strain curve and the shape of each curve is similar to the form of the power function, the power function is used to describe its stress–strain relationship. The constant term of this power function is “$\beta \cdot \sigma_{Y0}$”, which is used to describe the influence of the Bauschinger effect on the compression yield point. In order to facilitate a comparison of the fitting effect, the unloaded plastic section was also fitted using the equation proposed by Parker A.P.

The equations used in the constitutive model are shown in Equations (2)–(12). For the functions in each of the following regions (a)–(d), the functions of (1) Parker A.P.’s method are from Parker A.P.’s research, while the functions of (2) the power function method are formed based on the expression form of Parker A.P.’s but taking the shape of the data into consideration.

(a) Loading elastic region:

(2) $$E_{L}=\mathrm{constant},\;\sigma_{Y0}=\mathrm{constant}$$

where *E_L_* is the loading elasticity modulus and $\sigma_{Y0}$ is the loading yield stress.

(b) Loading plastic region:

(1) Parker A.P.’s method:

(3) $$\sigma_{L}^{p}=\sigma_{Y0}\left[1+a_{1}\cdot \tanh\left(c_{1}\cdot \varepsilon_{L}^{p}\right)+d_{1}\cdot 100\cdot \varepsilon_{L}^{p}\right]$$

(2) Power function method:

(4) $$\sigma_{L}^{p}=\sigma_{y0}+a_{2}\cdot {\left(100\cdot \varepsilon_{L}^{p}\right)}^{c_{2}}$$

where $\sigma_{L}^{p}$ is the loading plastic stress, $\varepsilon_{L}^{p}$ is the loading plastic strain, and *a*_1_, *c*_1_, *d*_1_, *a*_2_, and *c*_2_ are constants obtained through fitting.

(c) Unloading elastic region:

The functions of the compression part are related to the maximum tensile plastic strain $\varepsilon_{p}^{t}$ obtained above.

(1) Parker A.P.’s method:

(5) $$E_{UL}=E_{L}\cdot \left[1-m_{1}\tanh\left(n_{1}\times 100\varepsilon_{p}^{t}\right)\right]$$

(2) Power function method:

(6) $$E_{UL}=E_{L}\cdot \left[1-m_{2}\cdot {\left(\varepsilon_{p}^{t}\right)}^{n_{2}}\right]$$

where *E*_UL_ is the unloading elasticity modulus, while *m*_1_, *n*_1_, *m*_2_, and *n*_2_ are constants obtained through fitting.

(d) Unloading plastic region:

(1) Parker’s method:

(7) $$\sigma_{UL}^{p}=\sigma_{Y0}\left[\left(1+a_{1}-\beta \right)\cdot \tanh\left(\gamma \cdot 100\cdot \varepsilon_{UL}^{p}\right)+\beta +d_{1}\cdot \varepsilon_{UL}^{p}\right]$$

where *β* is the Bauschinger coefficient factor and *γ* is the unloading factor, which are described as follows:

(8) $$\beta =\left\{\begin{array}{ll} p_{1}\cdot {\left[\tan\left(1-\frac{1}{r_{1}}\cdot \varepsilon_{p}^{t}\right)\right]}^{q}+\beta_{0} & ,\;\;\;\;\;\;\;\mathrm{if}\;\;\;\;0<\varepsilon_{p}^{t}<r_{1}\\ \beta_{0} & ,\;\;\;\;\;\;\;\mathrm{if}\;\;\;\;\varepsilon_{p}^{t}>r_{1} \end{array}\right.$$

(9) $$\gamma =v_{1}\cdot {\left(100\cdot \varepsilon_{p}^{t}\right)}^{w_{1}}$$

where *p*_1_, *r*_1_, *q*, *β*_0_, *v*_1_, and *w*_1_ are constants obtained through fitting.

(2) Power function method:

(10) $$\sigma_{UL}^{p}=\beta \cdot \sigma_{Y0}+A\cdot {\left(\varepsilon_{UL}^{p}\right)}^{B}$$

where *β* is the Bauschinger coefficient factor, which is consistent with the definition above. *A* is the unloading multiple factor and *B* is the unloading power factor, which are described as follows:

(11) $$A=A_{a}+A_{b}\cdot {\left(100\cdot \varepsilon_{p}^{t}\right)}^{A_{c}}$$

(12) $$B=B_{a}B_{b}\cdot {\left(100\cdot \varepsilon_{p}^{t}\right)}^{B_{c}}$$

where *A_a_*, *A_b_*, *A_c_*, *B_a_*, *B_b_*, and *B_c_* are constants obtained through fitting.

With the functions and parameters above, the elastoplastic stress–strain relationship can be described.

### 3.3. Discussion of the Fitting Results

Based on the above equations, *Origin* software was used to perform function fitting for each region separately; the results obtained were as follows:

(a) Loading elastic region:

It is not necessary to fit the loading elastic region. Only the stress and strain at the tensile yield point need to be determined.

**Figure 5:**
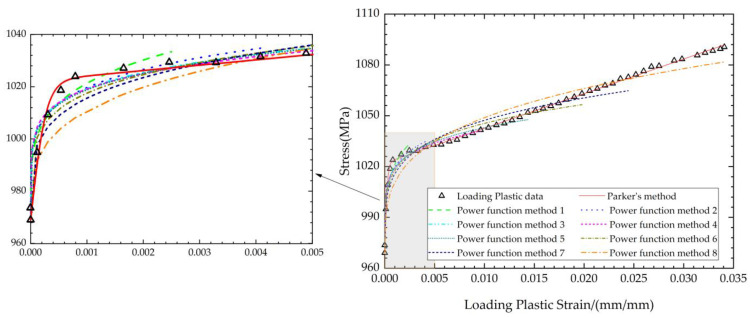
Fitting of the loading plastic curves with different methods.

(b) Loading plastic region:

Figure 5 shows the fitting of the loading plastic curves with different methods. As shown in Figure 5, Parker A.P.’s fitting method fits the loading elastic region very well. eight groups of stress–strain data with different maximum plastic strain are fitted with the power function method, and it is clear that the use of power functions cannot achieve the same level of accuracy as Parker A.P.’s fitting method in any of the cases. In fact, the shape of the power function is not suitable for fitting stress–strain data like these. Consequently, for the tensile plastic section, it is recommended to employ Parker A.P.’s method to describe its mechanical behavior.

**Figure 6:**
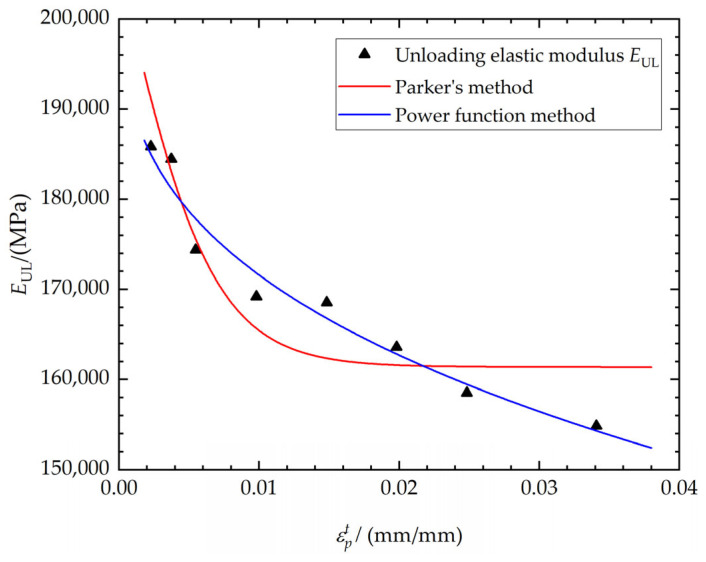
Fitting comparison of the unloading elastic modulus *E_UL_* and $\varepsilon_{p}^{t}$.

(c) Unloading elastic region:

The unloading elastic modulus *E_UL_* is a function of the loading elastic modulus *E_L_*. Figure 6 shows the fitting comparison of the unloading elastic modulus *E_UL_* and $\varepsilon_{p}^{t}$. As shown in Figure 6, the fitting of the power function method is more closely aligned with the experimental results, whereas Parker’s fitting method is less effective in capturing the relationship at higher tensile plastic strain.

**Figure 7:**
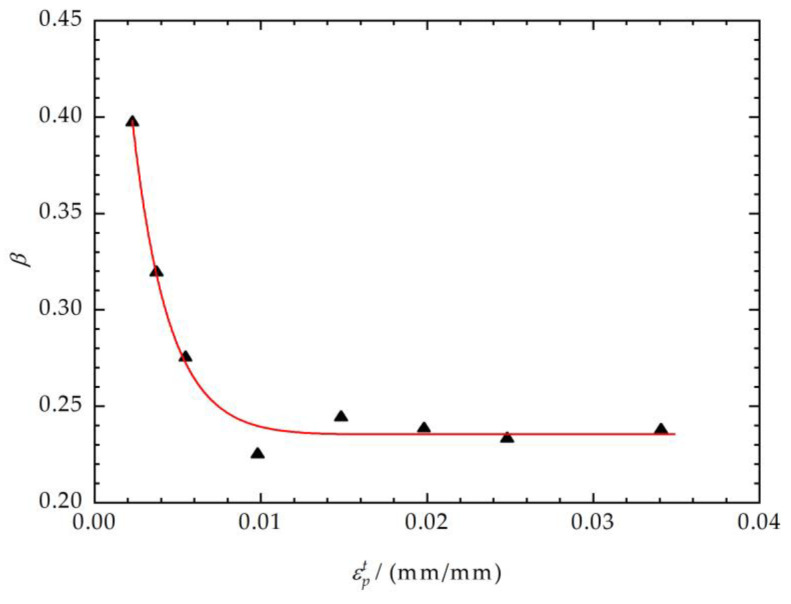
Fitting of the Bauschinger coefficient factor *β* and $\varepsilon_{p}^{t}$.

(d) Unloading plastic region:

The function fitting relationship between the Bauschinger coefficient factor *β* and loading yield stress $\varepsilon_{p}^{t}$ is shown in Figure 7. The unloading yield point, as the endpoint of the unloading elastic region, is described in the equation by *β* and $\sigma_{Y0}$. The Bauschinger coefficient factor *β* remains essentially unaltered when $\varepsilon_{p}^{t}$ is greater than 0.02.

**Figure 8:**
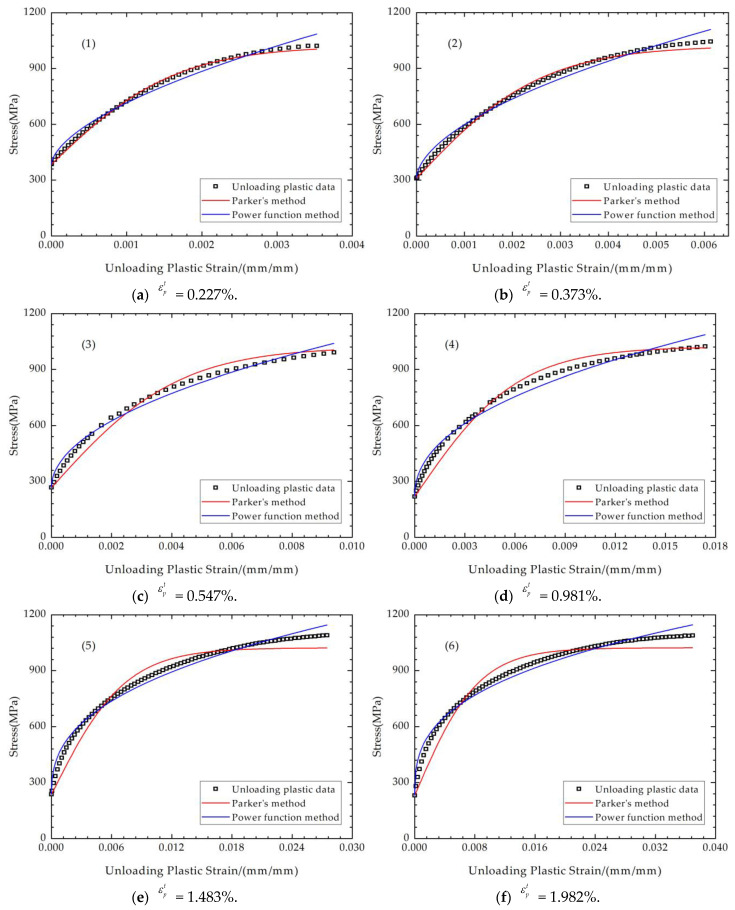

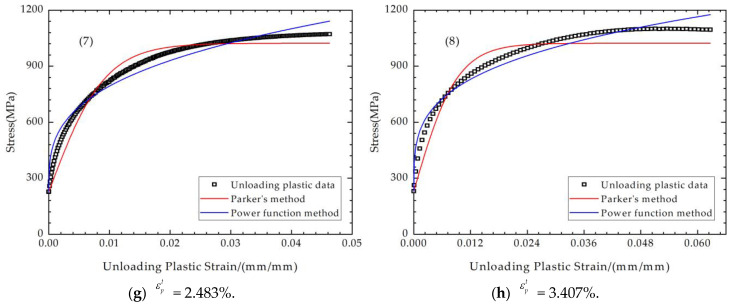
Fitting of the unloading plastic curves (1)–(8).

For the stress–strain relationship in the unloading plastic region, the two methods listed above were used to fit the eight groups of data, which started at the unloading yield point. The adjusted *R*^2^ was used as the evaluation standard to compare the accuracy of the fitting results of the two functions. The data on the horizontal axis are the unloading plastic strain, and the data on the vertical axis are the corresponding absolute values of true stress, as the Mises yield model was adopted in this paper. The fitting situations of the eight groups of unloading plastic data are shown in Figure 8, and the adjusted *R*^2^ of the different fitting methods is shown in Table 4. It can be seen from the results that, when $\varepsilon_{p}^{t}$ is small, there is not much difference in the fitting of Parker’s method and the power function method; when $\varepsilon_{p}^{t}$ is larger, the power function method shows better correlation. The average value of adjusted *R*^2^ also indicates that the power function method has a better fitting performance.

### 3.4. Power Function Kinematic Hardening Model of 35CrNi3MoVR Steel

Based on the analysis above, the fitting functions and their parameters were determined, and the elastoplastic expression best suited to describe 35CrNi3MoVR steel was concluded to be the power function kinematic hardening model. A summary of the final fitting model is provided in Table 5.

## 4. Finite Element Method of the Elastoplastic Constitutive Model

The elastoplastic model of the material was represented using ABAQUS UMAT, and the corresponding functions were generated in FORTRAN, with the assistance of the UHARD subroutine for the plastic calculations. In order to perform the numerical calculations from loading to unloading, it is necessary to determine the state of the element. After that, the determination of whether or not the element is in the plastic condition should be carried out. ABAQUS/CAE Standard implicit calculation was adopted for the FEM calculations, and a mesh model composed of one element was used, while C3D8 was chosen as the element type.

**Figure 9:**
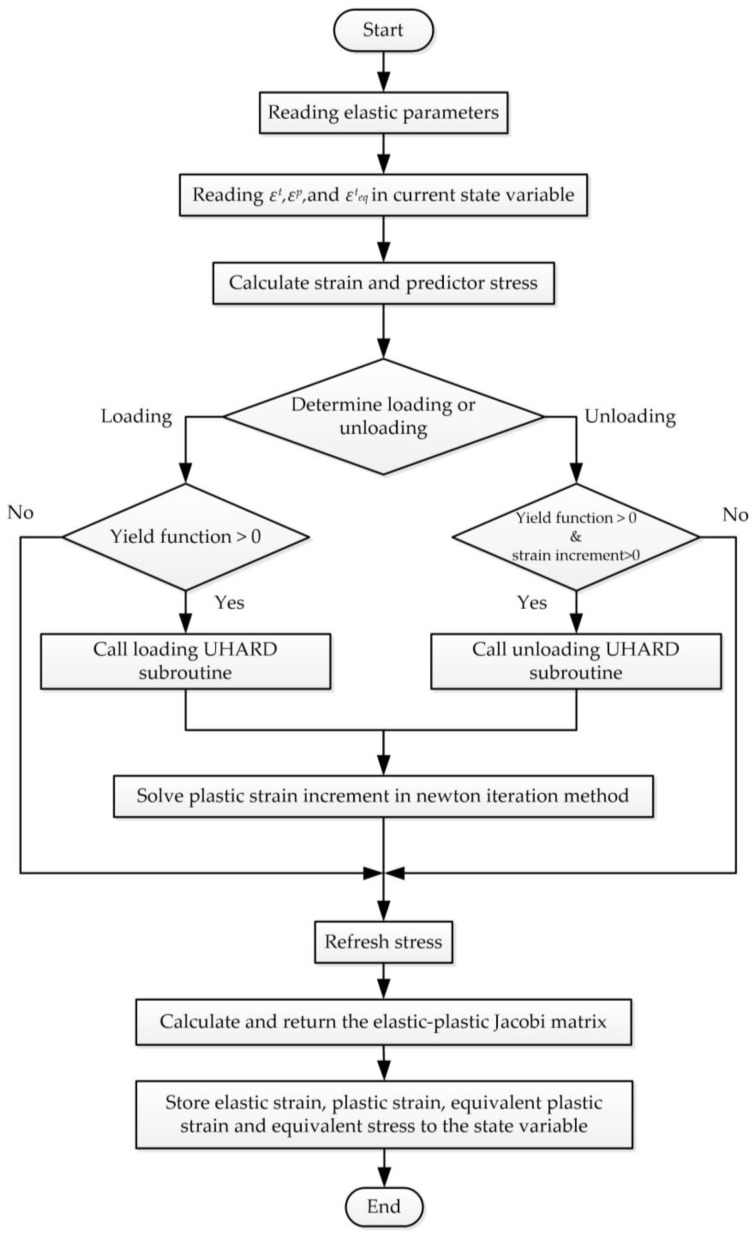
Logic of the finite element calculation.

The logic of the finite element calculation is shown in Figure 9. The direction factor *loaddir* is employed to indicate whether the element is loading or unloading. The judgment logic is as described in Equation (13):

(13) $$\left\{\begin{array}{rr} \mathrm{if}\;\varepsilon_{eqv,i+1}-\varepsilon_{eqv,i}>0\;\mathrm{and}\;\mathrm{sign}\left(\varepsilon_{1,i+1}-\varepsilon_{1,i}\right)\cdot \mathrm{sign}\left(\varepsilon_{2,i+1}-\varepsilon_{2,i}\right)\cdot \mathrm{sign}\left(\varepsilon_{3,i+1}-\varepsilon_{3,i}\right)>0\;,\;\mathrm{Then}\;loaddir & >0\\ \;\mathrm{else}\;\;\;\;\;\;\;\;\;\;\;\;\;\;\;\;\;\;\;\;\;\;\;\;\;\;\;\;\;\;\;\;\;\;\;\;\;\;\;\;\;\;\;\;\;\;\;\;\;\;\;\;\;\;\;\;\;\;\;\;\;\;\;\;\;\;\;\;\;\;\;\;\;\;\;\;\;\;loaddir & <0 \end{array}\right.$$

where the function *sign*( ) is a function that takes the symbol of the variants; $\varepsilon_{eqv}$ is the equivalent strain; $\varepsilon_{1}$, $\varepsilon_{2}$, and $\varepsilon_{3}$ are three normal stresses; and the subscripts *i* and *i*+1 represent the current calculation step and the next step, respectively. 

The yield can be determined through Equation (14):

(14) $$\left\{\begin{array}{rr} \mathrm{Yield}\;\mathrm{function}>0\;\;\;\;\;\;\;\;\;\;\;\;\;\;\;\;\;\;\;\;\;\;\;\;\;\;\;\;\;\;\;\;\;\;\;\;\;\;\;\;\;\;\;\;\;\;\;\;\;\;\;\;\;\;\;\;\;\;\;\;\;\;\;\;\; & ,\;\mathrm{if}\;loaddir>0\\ \mathrm{Yield}\;\mathrm{function}>0\;\mathrm{and}\;\varepsilon_{eqv\_i+1}-\varepsilon_{eqv\_i}>0\;\;\;\;\;\;\; & ,\;\mathrm{if}\;loaddir<0 \end{array}\right.$$

**Figure 10:**
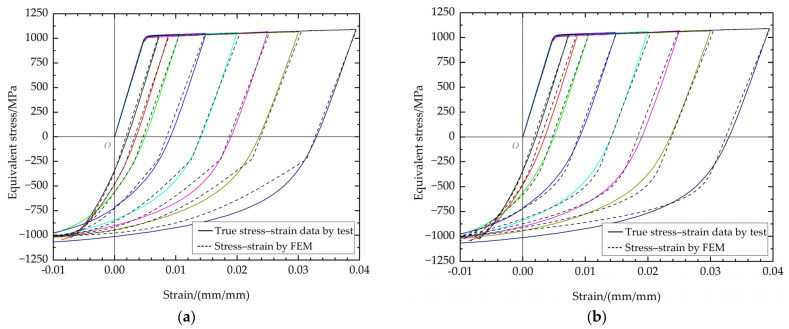
Comparison of the FEM results of Parker’s method (**a**) and the power function kinematic hardening model (**b**).

The results obtained from Parker’s fitting equation, as well as those from this study, are depicted in Figure 10. It can be observed that both methods effectively describe the Bauschinger effect and demonstrate the influence of tensile plastic strain on the compressive stress–strain curve. Nevertheless, it is well known that the reverse yield percentage is not too large for the autofrettage unloading process, so the fitting of the region near the unloading plastic point should be focused on. It is evident that the present study’s fitting equation exhibits a more precise agreement with the experimental data in these areas. For the loading regions (a) and (b), the stress–strain relationship calculated by FEM is in good agreement with the experimental data. This is because the equations used are consistent and suitable. However, for the unloading regions (c) and (d), the difference in the accuracy of different fitting equations comes from the difference in fitting degrees. The errors come from the fitting of the unloading elastic modulus. The fitting degree of *E*_UL_ by Parker’s method is not very satisfactory, which will to some extent affect the strain magnitude of the compressive plastic point, which is the starting point of the unloading plastic section (d). The lower fitting degree of the unloading plastic section further amplifies the disadvantage of Parker’s method in terms of accuracy, which will further deviate from the stress–strain relationship determined by the experiment. At the same time, the error of the stress–strain curve of the unloading plastic zone obtained by the two methods is also consistent with the fitting error in Figure 8a–h.

## 5. Implementation of Experimental Research and Finite Element Method Calculation

### 5.1. Introduction of the Autofrettage Experiment

**Figure 11:**
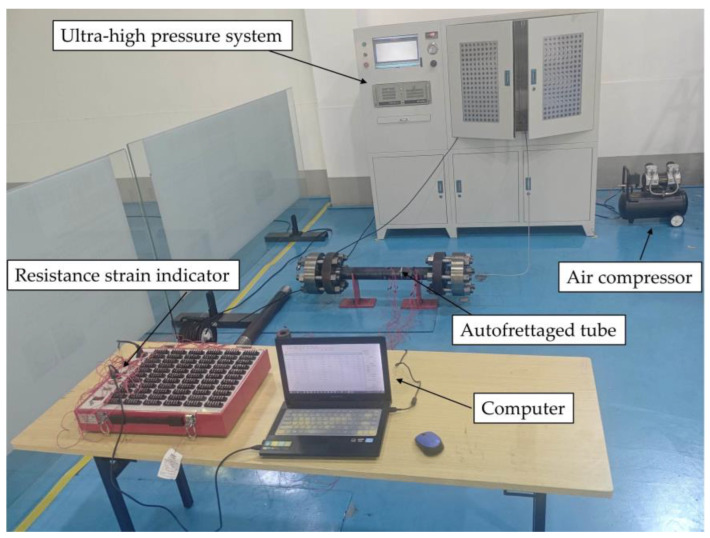
Scene of autofrettage experiment.

Experiments were conducted to investigate the hydraulic autofrettage of thick-walled straight tubes. Due to space constraints, only a brief description of the experimental process will be provided here. The experiment was conducted on two thick-walled cylinders, both with an outer diameter of 88 mm, an inner diameter of 40 mm, and a length of 900 mm (including threads at both ends). Both ends of the thick-walled tubes were sealed with flanged compression spherical lens gaskets. The autofrettage pressures were 680 MPa and 780 MPa, respectively, which were higher than the optimal autofrettage pressure of 631 MPa determined from the ideal elastoplastic model based on Mises stress according to theoretical calculations [40]. This pressure was considered to be less effective than expected for autofrettage in practice, due to the fact that the material’s tensile nonlinearities were not taken into account. After the autofrettage treatment, one end of the tube was cut off at a length of 25 cm, which was then used to detect the residual stresses in the radial and hoop directions by the Sachs method—a destructive residual stress detection method that is widely used in the detection of residual stresses of autofrettaged thick-walled tubes [41,42,43]. The sampling density was initially greater at the plastic zone than the elastic zone. A photograph of the working state of the experimental system is shown in Figure 11.

### 5.2. Issues with Autofrettage Calculation by the Finite Element Method

The autofrettage of 35CrNi3MoVR thick-walled cylinders was calculated in ABAQUS/CAE2021 using the finite element method. To compare the calculation results of different fitting models, the following models were employed in the present study: (i) the power function kinematic hardening model, hereafter referred to as the power function model; (ii) the revised kinematic hardening model proposed by Parker A.P., hereafter referred to as Parker’s model; and (iii) the elastoplastic model proposed by Huang [20], which was calculated with the elastoplastic properties of the material at the maximum tensile strain in the autofrettage process, hereafter referred to as Huang’s model. The results of these models were then compared with those of the ideal elastoplastic model, hereinafter referred to as the ideal E-P model. It should be noted that the simplified assumption of Huang’s model was employed, rather than the full adoption of its fitting and calculation method, which is based on the aforementioned assumption to degrade the model to the elastoplastic curve corresponding to the maximum tensile plastic strain.

**Figure 12:**
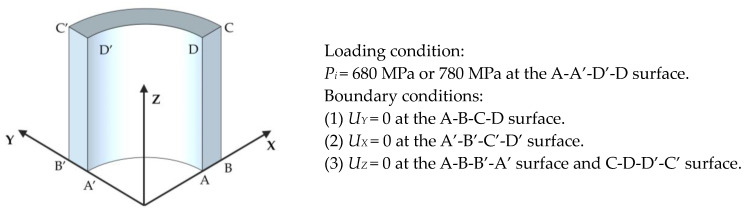
Loads and constraints of the 3D model for the hydraulic autofrettage process.

The finite element calculation utilized a three-dimensional solid model with dimensions equivalent to those of the thick-walled straight tubes employed in the experiment. Each tube can be regarded as a thick-walled straight tube with two open ends, allowing for an analysis of its mechanical properties. The modeling requires only one-quarter of the thick-walled straight tube and half of the actual tube length. ABAQUS/CAE Standard implicit calculation was adopted for the FEM calculations, and C3D8 was chosen as the element type, with a total number of 10,560. There were 22 elements in the thickness direction of the tube with an eccentricity of six, and 12 elements in the hoop direction of the quarter-circle, with 40 elements in the lengthwise direction. For the constraint and load settings, pressure was loaded on the inner surface of the tube, and normal constraints were applied to all symmetric surfaces. Subsequently, the internal pressure was first applied to the autofrettage pressure of 680 MPa or 780 MPa on the inner surface, and then unloaded to 0. The constraint and load conditions of the model are shown in Figure 12.

**Figure 13:**
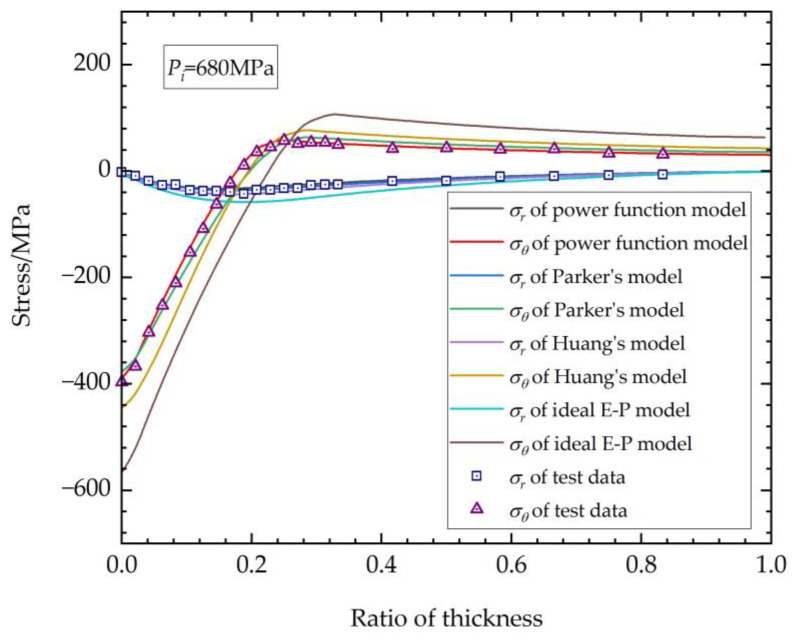
Residual stress after autofrettage (*P_i_* = 680 MPa).

**Figure 14:**
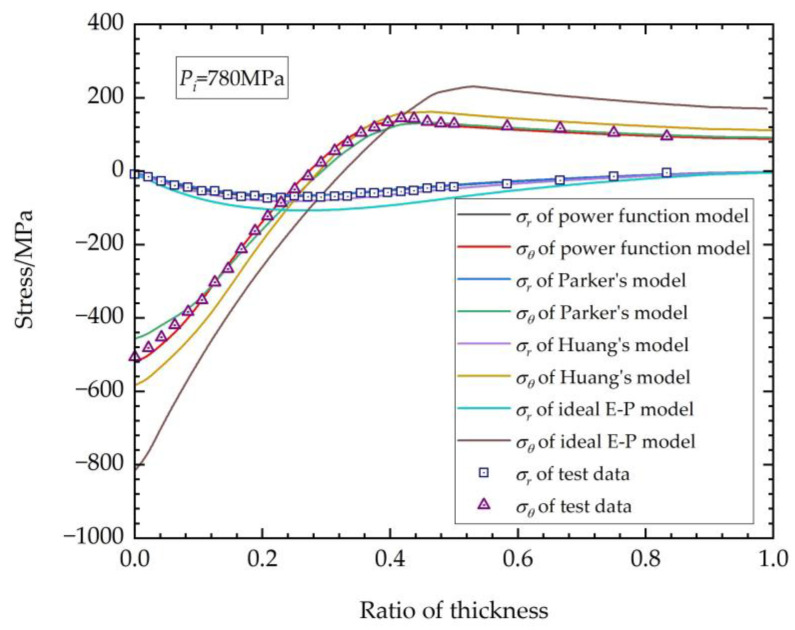
Residual stress after autofrettage (*P_i_* = 780 MPa).

The hoop stress $\sigma_{\theta }$ and radial stress $\sigma_{r}$ of the thick-walled straight tube after autofrettage, as obtained from the experiment, were plotted together with the FEM calculation results of the four models mentioned above, as shown in Figure 13 and Figure 14. Figure 13 depicts the results with an autofrettage pressure of 680 MPa, while Figure 14 shows the results for 780 MPa. Certain curves are largely on top of one another and not visible in the elastic zone of the tubes because the data calculated via different methods are close, which was caused by the common elastic and plastic loading functions.

## 6. Discussion

Form the results above, it is evident that the autofrettage effect calculated using the ideal E-P model is considerably higher than the experimentally measured values, and it is also significantly higher than the calculated results of other material elastoplastic models. This implies that the autofrettage calculation based on the ideal E-P model will overestimate the performance of autofrettage. It is also notable that there are discernable differences in the outcomes of the three models based on real elastoplastic properties.

The power function model is a modification of Parker’s model, considering the real stress–strain relationship characteristics of the studied material. For the power function model and Parker’s model, it can be seen from Figure 10 that, in fact, there is no discernible difference between the results of their FEM calculations in the loading regions, causing the plotted curves to exhibit a high degree of overlap at the elastic zone in Figure 13 and Figure 14. However, when the focus is shifted to the unloading plastic region, where the region is in proximity to the inner surface of the thick-walled tube, some intriguing phenomena emerge. The hoop stresses obtained by Parker’s model exhibit a more pronounced flattening change trend at the region near the inner surface. This discrepancy can be attributed to the fact that, when the strain material data with lower maximum tensile plastic strain are incorporated into Parker’s model, there is a lack of continuity between the compressive plastic and compressive elastic regions, as illustrated in Figure 10. The significant discrepancies in the fitting process result in errors in the subsequent FEM autofrettage calculations. It is evident that the utilization of the power function model also results in an underestimation or overestimation of the degree of the stress transitions in the position of the compressive plastic surface. Nevertheless, a comparison with the experimental data reveals that the calculation accuracy of the power function model is more satisfactory than that of Parker’s model, and there is greater advantage for the autofrettage FEM calculations using the power function method.

The results of the calculations using Huang’s model show higher accuracy than those of the ideal elastoplastic model, but with a tendency to overestimate the stress in the unloading zone compared with the results of the power function model and Parker’s model. In Huang’s model, the original appearance of the method is in the form of a double power function. The function structure is relatively simple, and the analytical solution can be obtained using theoretical methods. The application of Huang’s model necessitates the acquisition of a multitude of elastoplastic mechanical data under deferent tensile strains, a process that is inherently challenging in practice. However, according to the stress–strain model obtained above, the mechanical properties of the material under any tensile strain can be determined relatively accurately by degradation of the equations. This indicates that Huang’s model is just the downscaling of the power function model or Parker’s model.

As the most important part of the thick-walled structure bearing ultra-high inner pressure, the Mises stress results at the inner surface are listed in Table 6. The error of Mises stress calculated by the different models compared with the experimental results was adopted as the flag parameter for accuracy.

As can be seen from the table, the ideal E-P model has the worst precision, as it overestimates the stress at the inner surface by over 40%. The result of Huang’s model is much better than that of the ideal E-P model, with error over ~10%, but it is still higher than that of the kinematic hardening models. It can clearly be seen that the accuracy of the power function model used in this study is much greater than that of any other models, possessing the highest accuracy, with error of less than 3%, decreasing the error by at least 50% than other models.

The above results show that the revision of the kinematic hardening model offers great advantages in describing the elastoplastic mechanical properties of ultra-high-strength steels, and the accurate fitting of the uniaxial tensile and compressive mechanical test data is the key point of this technology, as the choice of the fitting functions plays the most important role in the whole process. A proper fitting method will lead to accurate autofrettage calculation results; this is particularly important in the research and design of ultra-high pressure vessels. 

## 7. Conclusions

In this paper, a study of the tensile–compression elastoplastic mechanical properties and Bauschinger effect of 35CrNi3MoVR material is presented, which was achieved by carrying out uniaxial tensile–compression tests. The processing method of the experimental data was presented, and an improved kinematic hardening model based on the power function and equations suitable for 35CrNi3MoVR material was constructed. The uniaxial tensile finite element calculation results demonstrate that the proposed model corresponds to the experimental data. The accuracy and applicability of the proposed elastoplastic intrinsic model of the material were further demonstrated by comparing the results of the hydraulic autofrettage tests of thick-walled straight tubes with those of the finite element calculations.

The following conclusions can be derived from the research presented in this paper:

(1) Uniaxial tensile and compression testing is a straightforward and effective method of investigating the compressive elastoplastic mechanical properties of materials, and it is generally accurate to use these test results for autofrettage calculation.

(2) For different materials, the fitting functions utilized in the kinematic hardening model to describe their tensile and compressive elastoplastic mechanical properties should still be adjusted according to their characteristics, even for similar materials.

(3) The results of autofrettage calculation using the ideal elastoplastic model will overestimate the effect of autofrettage, and the results calculated using the suitable corrected model obtained by fitting the actual elastoplastic tensile curve are more accurate than those calculated using the ideal elastoplastic model.

(4) For autofrettage calculation considering the Bauschinger effect, if the results of the calculation are based on the elastoplastic curve at the maximum tensile strain, they will not overestimate the autofrettage effect. However, this approach will result in a greater stress on the inner surface. Furthermore, it is challenging to obtain an accurate elastoplastic mechanical curve under the tensile overstrain in practical operations. It is possible to first obtain the corresponding elastoplastic relationship changes with the maximum tensile plastic strain and then degrade them to obtain the desired material data at the specific maximum tensile strain.

## Figures and Tables

**Table 1:** Chemical composition of 35CrNi3MoVR.

Element	C	Mn	Si	Cr	Ni	Mo	V	S
Standard weight percent/%	0.30~0.40	0.20~0.80	0.10~0.35	0.50~1.20	2.50~3.30	0.40~0.70	0.10~0.25	≤0.005
Test weight percent/%	0.38	0.47	0.18	1.07	2.84	0.50	0.17	0.0009

**Table 2:** Basic mechanical properties of 35CrNi3MoVR.

Yield Stress Rp0.2/MPa	UTS Rm/MPa	Elongation A/%	Young’s Modulus E/MPa	POISSON’S RATIO
≥960	1070~1230	≥16	206,000	0.3

**Table 3:** Tensile strain used in uniaxial tensile–compression tests.

No.	1#	2#	3#	4#	5#	6#	7#	8#
Nominal total tensile strain	0.7%	0.85%	1.0%	1.50%	2.0%	2.5%	3.0%	4.0%
Tensile plastic strain	0.227%	0.373%	0.547%	0.981%	1.483%	1.982%	2.483%	3.407%

**Table 4:** Adjusted *R*^2^ of different fitting methods.

$\varepsilon_{p}^{t}$	0.227%	0.373%	0.547%	0.981%	1.483%	1.982%	2.483%	3.407%	Average (%)
Parker A.A.	0.99915	0.99813	0.99323	0.99341	0.99159	0.99030	0.98980	0.98797	0.99295
Power function	0.99521	0.99571	0.99587	0.99488	0.99652	0.99626	0.99007	0.99378	0.99479

**Table 5:** Expressions of various regions obtained from fitting.

Region	Function	Parameter
(a)	$E_{L}=\mathrm{constant},\;\sigma_{Y0}=\mathrm{constant}$	$E_{L}=205976.5\;\mathrm{MPa},\;\sigma_{Y0}=969.0662\;\mathrm{MPa}$
(b)	$\sigma_{L}^{p}=\sigma_{Y0}\left[1+a\cdot \tanh\left(c\cdot \varepsilon_{L}^{p}\right)+d\cdot 100\cdot \varepsilon_{L}^{p}\right]$	a=0.0548, c=3622.8, d=0.0211
(c)	$E_{UL}=E_{L}\cdot \left[1-m\cdot {\left(\varepsilon_{p}^{t}\right)}^{n}\right]$	m=0.7713, n=0.3324
(d)	$\sigma_{UL}^{p}=\beta \cdot \sigma_{Y0}+A\cdot {\left(\varepsilon_{UL}^{p}\right)}^{B}$	$\beta =\left\{\begin{array}{cc} 0.0637\cdot {\left[\tan\left(1-50\cdot \varepsilon_{p}^{t}\right)\right]}^{q}+0.2355 & ,\;\;\mathrm{if}\;\;\;\;0<\varepsilon_{p}^{t}<0.02\\ \;\;\;\;\;\;\;\;\;\;\;\;\;\;\;\;\;\;\;\;\;\;\;\;\;\;\;\;\;\;\;\;\;\;\;\;\;\;\;0.2355\;\;\;\;\;\;\;\;\;\;\;\; & ,\;\;\mathrm{if}\;\;\;\;\;\;\;\;\;\;\varepsilon_{p}^{t}>0.02 \end{array}\right.$ $A=492.9359+187.3760\cdot {\left(100\cdot \varepsilon_{p}^{t}\right)}^{-1.0029}$ $B=1.0816-0.6404\cdot {\left(100\cdot \varepsilon_{p}^{t}\right)}^{0.1920}$

**Table 6:** Mises stress at the inner surface calculated by different models.

Model	Autofrettage Pressure/MPa	Mises Stress/MPa	Error
Experimental result	680	351.43	-
Power function model		344.14	2.07%
Parker’s model		332.56	5.37%
Huang’s model		391.52	11.41%
Ideal E-P model		499.45	42.12%
Experimental result	780	443.41	--
Power function model		452.21	1.98%
Parker’s model		396.40	10.60%
Huang’s model		507.02	14.35%
Ideal E-P model		713.07	60.82%

## Data Availability

The data presented in this study are available upon request from the corresponding author, due to privacy.

## References

[1] Rees D.W.A. (1991). The Fatigue Life of Thick-Walled Autofrettaged Cylinders with Closed Ends. Fatigue Fract. Eng. Mater. Struct..

[2] Alegre J.M., Bravo P., Preciado M. (2006). Design of an Autofrettaged High-Pressure Vessel, Considering the Bauschinger Effect. Proc. Inst. Mech. Eng. Part E J. Process Mech. Eng..

[3] Dixit U.S., Shufen R., Silberschmidt V.V. (2020). 2—Finite Element Method Modeling of Hydraulic and Thermal Autofrettage Processes. Mechanics of Materials in Modern Manufacturing Methods and Processing Techniques.

[4] Davidson T.E., Barton C.S., Reiner A.N., Kendall D.P., Rossi B.E. (1963). Overstrain of High-Strength Open-End Cylinders of Intermediate Diameter Ratio. Experimental Mechanics.

[5] Shufen R., Dixit U.S. (2018). A Review of Theoretical and Experimental Research on Various Autofrettage Processes. J. Press. Vessel Technol..

[6] Hu Z., Parker A.P. (2019). Swage Autofrettage Analysis—Current Status and Future Prospects. Int. J. Press. Vessel. Pip..

[7] Perl M., Perry J., Aharon T., Kolka O. (2012). Is There an “Ultimate” Autofrettage Process?. J. Press. Vessel Technol..

[8] Roy A.K., Kamal S.M., Patil R.U., Rao V.V., Dixit U.S., Echempati R., Dey S. (2023). Practicing Hydraulic Autofrettage for Strengthening a Gun Barrel: Critical Issues and Challenges. Engineering Pedagogy: A Collection of Articles in Honor of Prof. Amitabha Ghosh.

[9] Vullo V., Vullo V. (2014). Thick-Walled Circular Cylinders in the Linear Elastic-Perfectly Plastic State After Loading Beyond the Elastic Range. Circular Cylinders and Pressure Vessels: Stress Analysis and Design.

[10] Alexandrov S., Lyamina E., Aoh J.-N., Jeng Y.-R. (2020). Description of Residual Stresses in Autofrettaged Open-Ended Cylinders Made of High-Strength Steel. Materials.

[11] Shim W.S., Kim J.H., Lee Y.S., Cha K.U., Hong S.K. (2010). A Study on Hydraulic Autofrettage of Thick-Walled Cylinders Incorporating Bauschinger Effect. Exp. Mech..

[12] Çandar H., Filiz İ.H. (2017). Experimental Study on Residual Stresses in Autofrettaged Thick-Walled High Pressure Cylinders. High Press. Res..

[13] Ma Y., Zhang S.Y., Yang J., Zhang P. (2022). Neutron Diffraction, Finite Element and Analytical Investigation of Residual Strains of Autofrettaged Thick-Walled Pressure Vessels. Int. J. Press. Vessel. Pip..

[14] Maleki M., Farrahi G.H., Haghpanah Jahromi B., Hosseinian E. (2010). Residual Stress Analysis of Autofrettaged Thick-Walled Spherical Pressure Vessel. Int. J. Press. Vessel. Pip..

[15] Chen P.C.T. (1986). The Bauschinger and Hardening Effect on Residual Stresses in an Autofrettaged Thick-Walled Cylinder. J. Press. Vessel Technol..

[16] Livieri P., Lazzarin P. (2001). Autofrettaged Cylindrical Vessels and Bauschinger Effect: An Analytical Frame for Evaluating Residual Stress Distributions. J. Press. Vessel Technol..

[17] Milligan R.V., Koo W.H., Davidson T.E. (1966). The Bauschinger Effect in a High-Strength Steel. J. Basic Eng..

[18] Megahed M.M., Abbas A.T. (1991). Influence of Reverse Yielding on Residual Stresses Induced by Autofrettage. Int. J. Mech. Sci..

[19] Troiano E., Parker A.P., Underwood J., Mossey C. (2003). Experimental Data, Numerical Fit and Fatigue Life Calculations Relating to the Bauschinger Effect in High Strength Armament Steels. J. Press. Vessel Technol..

[20] Huang X.P. (2005). A General Autofrettage Model of a Thick-Walled Cylinder Based on Tensile-Compressive Stress-Strain Curve of a Material. J. Strain Anal. Eng. Des..

[21] De Swardt R.R. (2006). Material Models for the Finite Element Analysis of Materials Exhibiting a Pronounced Bauschinger Effect. J. Press. Vessel Technol..

[22] Parker A.P. Assessment and Extension of an Analytical Formulation for Prediction of Residual Stress in Autofrettaged Thick Cylinders. Proceedings of the PVP2005 High Pressure Technology, Nondestructive Evaluation, Pipeline Systems, Student Paper Competition.

[23] Parker A.P. (2000). Autofrettage of Open-End Tubes—Pressures, Stresses, Strains, and Code Comparisons. J. Press. Vessel Technol..

[24] Parker A.P., Underwood J.H., Kendall D.P. (1999). Bauschinger Effect Design Procedures for Autofrettaged Tubes Including Material Removal and Sachs’ Method. J. Press. Vessel Technol..

[25] Parker A.P., Troiano E., Underwood J.H., Mossey C. (2003). Characterization of Steels Using a Revised Kinematic Hardening Model Incorporating Bauschinger Effect. J. Press. Vessel Technol..

[26] Faghih S., Jahed H., Behravesh S.B. (2018). Variable Material Properties Approach: A Review on Twenty Years of Progress. J. Press. Vessel Technol..

[27] Troiano E., Parker A.P., Underwood J.H. (2004). Mechanisms and Modeling Comparing HB7 and A723 High Strength Pressure Vessel Steels. J. Press. Vessel Technol..

[28] Farrahi G.H., Voyiadjis G.Z., Hoseini S.H., Hosseinian E. (2013). Residual Stress Analysis of the Autofrettaged Thick-Walled Tube Using Nonlinear Kinematic Hardening. J. Press. Vessel Technol..

[29] Troiano E., Underwood J.H., Parker A.P. (2006). Finite Element Investigation of Bauschinger Effect in High-Strength A723 Pressure Vessel Steel. J. Press. Vessel Technol..

[30] Gibson M.C. (2008). Determination of Residual Stress Distributions in Autofrettaged Thick Cylinders.

[31] Hu Z., Gibson M.C., Parker A.P. (2021). Swage Autofrettage FEA Incorporating a User Function to Model Actual Bauschinger Effect. Int. J. Press. Vessel. Pip..

[32] Hu Z., Puttagunta S. (2012). Computer Modeling of Internal Pressure Autofrettage Process of a Thick-Walled Cylinder with the Bauschinger Effect. Am. Trans. Eng. Appl. Sci..

[33] Loffredo M. (2018). Measurement and Modelling of Bauschinger Effect for Low-Level Plastic Strains on AISI 4140 Steel. Procedia Struct. Integr..

[34] Molaie M., Darijani H., Bahreman M., Hosseini S.M. (2018). Autofrettage of Nonlinear Strain-Hardening Cylinders Using the Proposed Analytical Solution for Stresses. Int. J. Mech. Sci..

[35] Li Y., Wang W., Pan M., Cao W., Ma X., Li Y. (2023). Fatigue Life Analysis of High-Pressure Seamless Steel Cylinder for Hydrogen Using Autofrettage Design. Int. J. Press. Vessel. Pip..

[36] Shufen R., Singh N.P., Dixit U.S. (2023). Thermally Assisted Rotational Autofrettage of Long Cylinders with Free Ends. J. Press. Vessel Technol..

[37] Hu Z., Parker A.P. (2023). Fluid End Blocks: Numerical Analysis of Autofrettage and Reautofrettage Based Upon a True Material Model. J. Press. Vessel Technol..

[38] (2017). Ultra-High Pressure Vessels General.

[39] Dunne F., Petrinic N. (2005). Introduction to Computational Plasticity.

[40] Zhu R., Yang J. (1998). Autofrettage of Thick Cylinders. Int. J. Press. Vessel. Pip..

[41] Sachs G. (1927). Der Nachweis Innerer Spannungen in Stangen Und Rohren. Zeits Zeitshrift Met..

[42] De Swardt R.R. (2003). Finite Element Simulation of the Sachs Boring Method of Measuring Residual Stresses in Thick-Walled Cylinders. J. Press. Vessel Technol..

[43] Parker A.P. (2004). A Critical Examination of Sachs’ Material-Removal Method for Determination of Residual Stress. J. Press. Vessel Technol..

